# A Dissertation on the Practice of Medicine, Containing an Account of the Causes, Symptoms, and Treatment of Diseases: And Adapted to the Use of Physicians and Families

**Published:** 1849-10

**Authors:** 


Art. VII.—*A Dissertation on the Practice of Medicine, containing an account of the causes, symptoms, and treatment of diseases: and adapted to the use of Physicians and Families.* By Tomlinson Fort, M. D. 1 vol. 8vo. pp. 740. Milledgeville, Ga.

A very queer volume, bearing the above title, has been laid upon our table. As the production of an *American Physician*, it was taken up with more than ordinary pleasure, and examined with more than ordinary care and attention. We are sorry to say, however, that we were disappointed in our pleasant anticipations, and but poorly repaid for our labor.

We do not, therefore, regret that our limited space prevents an extended review—the brevity of this notice bearing an accurate ratio to the real value of the work.

If our author had but reflected a moment before he engaged in his undertaking, he would certainly have seen that the space which he has allowed himself is entirely too small to contain more than the *merest fraction* of what is valuable in the Practice of Medicine, Materia Medica, Surgery, and so much of the Obstetric Art as is embraced in Diseases of Females—with a Lexicon superadded! especially when so much of that space—small as it is—is taken up by matter which would have appeared to more advantage in the "Funny" column of a country newspaper, than in a sober treatise of Practical Medicine.

Altogether aside, however, from this, we do not think that a work on the "*Practice of Medicine*," in which an actually greater space is devoted to *Mesmerism and Somnambulism*, than to Phthisis; or to the whole subject of Diseases of the Heart; or to Pneumonia and Pleurisy—which, by the way, the author, singularly enough, says cannot be distinguished from each other:—in which Auscultation is only mentioned to be derided, and Percussion is never once even hinted at: and in which the Hydriodate of Potash is said to be "*a tripple compound of* MERCURY, *Iodine and Potash;*" such a work, we say, we do not think will be consulted by experienced Physicians, with much expectation of benefit to be derived therefrom; nor can it be considered a very reliable guide for the student of medicine, or the "*dear people*" who figure so largely in its title-page and introduction.

There is one observation made by the author in his preface, which, for its modesty, deserves great commendation. "This work," says he, "is in its nature ephemeral; its author does not claim for it a place among the standard works of the day." We are glad to learn that this is the case, for it shows that he possesses one happy faculty not common to every writer, viz: a proper appreciation of the merits of his own labors.

We trust that Dr. Fort will allow us to assure him that this plan of making "every man his own doctor," (which is one of the designs of this volume) will not, and from the necessity of the case, cannot succeed: the attempt will never be productive of any good to the community at large, and must always redound, as in this case, to the discredit of the author—of whom we now take our leave with the kindest feelings, commending to his careful study, among other things, Materia Medica and Auscultation and Percussion. A more extended knowledge of both of these subjects will surely not injure his reputation, either as a practitioner, or as a writer on Practical Medicine.

